# Enriched Acoustic Environment Therapy (EAE): A Cost-Effective and Feasible Alternative to Tinnitus Retraining Therapy (TRT)

**DOI:** 10.3390/healthcare13243248

**Published:** 2025-12-11

**Authors:** Marta Fernández-Ledesma, Ricardo Sanz-Fernández, María Cuesta, Pedro Cobo

**Affiliations:** 1 Department of Medicine, Faculty of Biomedical and Health Sciences, European University of Madrid, C/Tajo s/n, 28670 Villaviciosa de Odón, Spain; ricardosanz.orl@gmail.com; 2 Institute for Physical and Information Technologies (ITEFI), Spanish National Research Council (CSIC), 28006 Madrid, Spain; m.cuesta@csic.es (M.C.); pedro.cobo@csic.es (P.C.)

**Keywords:** sound therapy, tinnitus, TRT, EAE, neurophysiological model, tinnitus retraining therapy, enriched acoustic environment, habituation, tele-audiology, feasibility, adherence

## Abstract

**Highlights:**

**What are the main findings?**

- EAE produced clinically meaningful and statistically significant tinnitus reduction (~50% improvement in THI and TFI) after just 1 h/day for 4 months.
- Both continuous and sequential EAE stimuli achieved comparable outcomes with very low non-response rates (3–8%), demonstrating robust and consistent clinical effectiveness.

**What are the implications of the main finding?**

- EAE achieved tinnitus improvements similar to those reported for TRT while re-quiring over 90% less listening time, offering markedly greater time efficiency and higher treatment feasibility.
- The simplicity, accessibility, and low resource requirements of EAE support its in-tegration into routine and public healthcare settings, including tele-audiology and remote care models.

**Abstract:**

**Background/Objectives:** Tinnitus affects approximately 15% of the population and lacks a universally effective treatment. Tinnitus Retraining Therapy (TRT) is widely used but requires 6–8 h of daily sound exposure for 1–2 years, limiting accessibility and adherence. This study evaluated the clinical feasibility and therapeutic effectiveness of Enriched Acoustic Environment therapy (EAE), a streamlined alternative using individualized sound stimulation with a markedly reduced treatment burden, and compared its time efficiency with published TRT outcomes. **Methods:** 82 adults with chronic tinnitus received standardized counseling and completed one of two EAE protocols (continuous or sequential). Participants listened to their personalized stimulus for 1 h/day over four months. Tinnitus severity (THI, TFI) and time-efficiency metrics (improvement per 10 listening hours) were assessed and compared with TRT studies reporting baseline and post-treatment THI. **Results:** EAE produced clinically relevant and statistically significant improvements, with 51.6% THI and 49.8% TFI reduction (*p* < 0.001). Both stimuli achieved similar outcomes with high responder rates. EAE yielded ~2.3 THI-point improvement per 10 h (~4.3% relative gain), demonstrating substantially greater time efficiency—approximately 20 times higher than values reported for standard TRT protocols. **Conclusions:** EAE achieved robust symptom reduction with dramatically lower treatment burden, high adherence, and strong clinical feasibility. These findings support EAE as an accessible, time-efficient alternative to TRT. Controlled long-term studies are warranted.

## 1. Introduction

Tinnitus is the perception of sound without an external source and affects roughly 15% of the population and causes severe distress in up to 2.5% of cases [1,2], representing a substantial public health concern. Its multifactorial origins involve both peripheral and central auditory mechanisms, often associated with sensorineural hearing loss, noise exposure, aging, and psychological factors. Maladaptive neuroplasticity and interactions with the limbic and autonomic systems contribute to the persistence and emotional burden of tinnitus [2,3]. Effective treatments must therefore address both perceptual and reactive components of the condition.

Among non-pharmacological interventions, TRT is one of the most established approaches. It combines counseling with sound therapy to facilitate habituation within the neurophysiological framework [2]. Although clinically effective, TRT requires 6–8 h of daily sound exposure for 1–2 years, typically through specialized devices. These demanding protocols can hinder accessibility, adherence, and large-scale implementation, particularly in public healthcare systems [4].

Despite its widespread clinical use and extensive supporting experience, TRT’s position within evidence-based practice frameworks has been questioned. Because TRT relies on the synergistic interaction between counseling and sound therapy, true blinding is not feasible, and designing an inert control condition is difficult, as many neutral sounds may exert therapeutic effects on their own [5]. As a result, modern evidence-grading systems often classify TRT studies as high or unclear risk of bias, not because TRT is ineffective, but because its design is incompatible with conventional methodological standards. As a result, TRT is frequently underrepresented in clinical guidelines, even though comparative studies have shown improvements equal to or superior to cognitive–behavioral therapy (CBT) [6,7,8]. This discrepancy highlights a key methodological gap: sound-based therapies may be effective, yet difficult to evaluate within traditional randomized, blinded frameworks.

Given these limitations, alternative evaluation metrics are needed. Time efficiency (relating clinical improvement to total therapeutic exposure time) may offer a more pragmatic assessment of real-world benefit, where treatment burden and adherence strongly influence outcomes.

Enriched Acoustic Environment therapy (EAE) was developed as a streamlined alternative to TRT, maintaining the same neurophysiological principles but drastically reducing treatment time. EAE delivers individualized, audiometry-shaped sound stimulation for one hour per day over four months, using standard headphones. Earlier feasibility studies report symptom reduction comparable to TRT, higher adherence, and lower cost and complexity [9,10,11,12,13].

This study evaluated the feasibility, adherence, and clinical outcomes of EAE and compared its time efficiency with published TRT protocols to determine whether effective habituation can be achieved with substantially lower sound-exposure demands.

## 2. Materials and Methods

This section describes the study design, participant characteristics, and procedures implemented to evaluate the clinical feasibility of EAE.

### 2.1. Participants

The study was approved by the Research Ethics Committee of the European University of Madrid (protocol code CIPI/23.009, approved on 30 January 2023) and conducted in accordance with the Declaration of Helsinki for research involving human subjects, as well as the Spanish Data Protection Law (RD1720/2007). All participants provided written informed consent prior to inclusion in the study.

Eligible participants were adults aged 18 years or older with chronic, non-pulsatile tinnitus and a Tinnitus Handicap Inventory (THI) score ≥ 20. Exclusion criteria included recent ear surgery, severe Ménière’s syndrome (or an episode within the preceding six months), hydrocephalus, or a Hospital Anxiety and Depression Scale (HADS-D) score ≥ 16.

A prospective experimental clinical feasibility study was conducted at the Audiology Laboratory of the European University of Madrid. Upon enrollment, participants underwent a baseline assessment, including pure-tone audiometry (air and bone conduction), tinnitus characterization (frequency and loudness), and completion of the Tinnitus Handicap Inventory (THI) and Tinnitus Functional Index (TFI) questionnaires. During this same visit, all participants attended a standardized counseling session.

**Figure 1:**
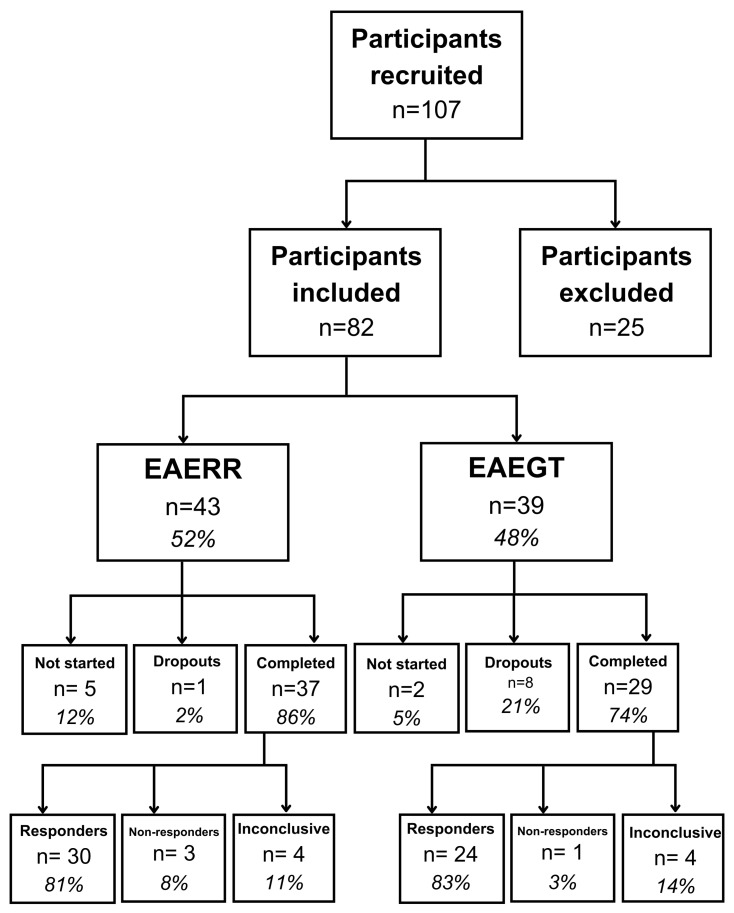
Flow diagram of participants included in this study. EAERR: enriched acoustic environment therapy with continuous sound; EAEGT: enriched acoustic environment therapy with sequential sound.

Following counseling, participants selected their preferred personalized auditory stimulus for therapy based on their personal comfort and auditory preferences. As described in Figure 1, a total of 82 individuals were included in the study: 43 participants chose the EAE continuous (EAERR) stimulus—6 (14%) dropped out, 5 of whom did not initiate therapy after counseling and 1 who withdrew during the intervention—while 39 participants selected the EAE sequential (EAEGT) stimulus, with 10 (26%) dropouts (2 did not begin therapy after counseling and 8 withdrew during treatment).

At the completion of the four-month intervention period, outcome measures (THI and TFI) were reassessed under the same conditions as baseline testing to evaluate post-treatment changes. In the EAERR group, 81% (*n* = 30) of participants demonstrated clinical improvement, reflected by a reduction in both THI and TFI scores. Three participants (8%) showed no improvement, while four participants (11%) exhibited inconclusive outcomes, with one index improving and the other worsening. Similarly, in the EAEGT group, 24 participants (83%) showed improvement, 1 participant (3%) showed no improvement, and 4 participants (14%) had inconclusive results.

Throughout the intervention, participants received monthly telephone follow-ups to monitor progress, reinforce adherence, and address any questions or difficulties related to the listening protocol. All self-report questionnaires were submitted electronically via email.

### 2.2. Hearing Levels

**Figure 2:**
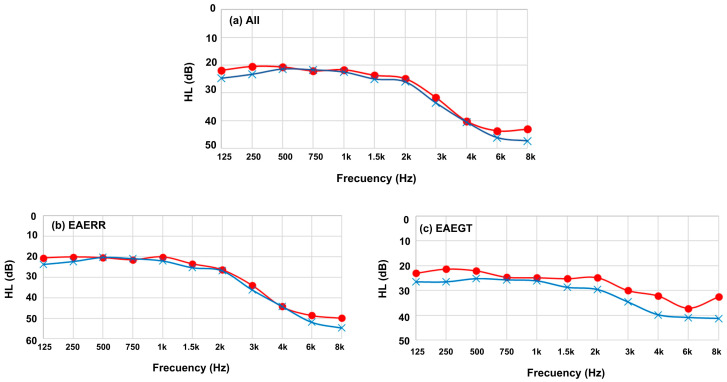
Average HL for the right ear (red) and left ear (blue): (**a**) all participants included in the study; (**b**) participants who selected the EAE continuous (EAERR) stimulus; and (**c**) participants who selected the EAE sequential (EAEGT) stimulus.

The EAERR and EAEGT sound therapies were specifically developed to provide individualized auditory stimulation based on each participant’s hearing loss profile [10]. Pure-tone audiometry was conducted in our laboratory for all participants, measuring thresholds across frequencies from 125 Hz to 8 kHz. The mean audiometric thresholds (hearing levels, HL) for the entire cohort, as well as stratified by sound therapy group, are presented in Figure 2. Pure-tone Average 4 (PTA4) values were 27.6 dB HL in the right ear and 29.3 dB HL in the left ear overall; in EAERR, 27.8/28.5 dB HL, and in EAETG, 26.1/30.6 dB HL.

### 2.3. Tinnitus Characteristics

The participants’ medical histories were obtained immediately after providing informed consent, using a standardized tinnitus characteristics questionnaire. The collected data included information on tinnitus attributes, such as temporal variability, perceived pitch or frequency, sound intensity, lateralization (bilateral, left ear, or right ear), and modulations related to neck, jaw, or head movements. Participants also reported the presumed etiology of their tinnitus, any previous treatments, and relevant comorbidities.

Table 1 presents the distribution of responding participants according to the selected sound therapy, stratified by gender, and reports the mean age (M), standard deviation (SD), tinnitus laterality, tinnitus duration, perceived tinnitus frequency, and baseline THI and TFI scores. Regarding tinnitus laterality, participants were assigned to bilateral (B), right ear (R), or left ear (L) groups based solely on their responses to an evaluation sheet. Therefore, an MRI examination to identify or exclude retrocochlear causes, as recommended by NICE guidelines [14], was not performed.

Participants also reported tinnitus etiologies and comorbidities. Hearing loss was the most common factor (≈90%), often alongside additional contributors. Stress was reported in 40% of cases, followed by sudden sensorineural hearing loss (9%), acoustic trauma (9%), COVID-19–related onset (7%), barotrauma (2%), among others. Comorbidities were frequent, with 63% of participants reporting anxiety and 19% reporting depression. Many cases were multifactorial or ultimately idiopathic.

### 2.4. Counseling and Sound Therapy

After providing informed consent and completing the audiometric assessment, participants attended a standardized counseling session, provided by MFL (audiologist), lasting approximately 60 min. During this session, a PowerPoint presentation outlined the auditory system’s function and the mechanisms, etiology, epidemiology, and treatment options for tinnitus, while also explaining the relationship between tinnitus, the limbic system, and the autonomic nervous system, with emphasis on the neurophysiological mechanisms underlying symptom perception and habituation.

Following counseling, participants selected their preferred auditory stimulus (EAERR or EAEGT). The stimulus EAEGT consists of a sequence of gammatones, given by the following [9]:

(1) $$\mathrm{EAEGT}\left(t\right)=\sum_{m}A_{m}\left(f_{m}\right)\left(\frac{b_{m}^{4}}{6}\right)t^{3}e^{-b_{m}\left(t-\tau_{m}\right)}cos\left[2\pi f_{m}\left(t-\tau_{m}\right)\right]\;$$

where the following is notable:

(2) $$A_{m}\left(f_{m}\right)=10^{\frac{HL\left(f_{m}\right)}{20}}$$

This is the amplitude of each gammatone, *HL*(*f_m_*) is the hearing loss at frequency *f_m_*, and *b_m_* is as follows:

(3) $$b_{m}=ERB=0.108f_{m}+24.7$$

This is the equivalent rectangular band (ERB), and *τ_m_* is the time rate of tones (number of pulses per second).

The equation for the stimulus EAERR is as follows [9]:

(4) $$EAERR\left(t\right)=F^{-1}\left\{A\left(f\right)RAND\left(f\right)\right\}$$

where f is the frequency (in the audio frequency range 125 Hz–8 kHz), *A*(*f*) is an amplitude factor given by the interpolation of Equation (2) in the full frequency range, *RAND*(*f*) is a random function, and $F^{-1}$ stands for inverse Fourier transform. Therefore, the EAERR is the implementation of the broadband stimulus matched to the hearing loss curve.

After that, they received detailed instructions on its use, including guidance on sound volume adjustment, headphone selection, and the recommended listening schedule. Selection was based solely on subjective comfort and sound preference; no audiometric or clinician-driven criteria were used. All participants followed the same listening protocol: one hour of daily exposure at their convenience for four consecutive months. In every case, the auditory stimuli were provided as MP3 audio files to be played on a personal device (e.g., smartphone, tablet, or MP3 player) connected to headphones. The listening intensity was set at the mixing point [2], defined as the level just below the perceived tinnitus loudness.

## 3. Results

After four months of sound therapy for one hour a day, the following results were obtained:

### 3.1. Clinical Outcomes Following EAE Therapy

EAE produced a clinically relevant and statistically significant reduction in tinnitus severity [15,16]. As shown in Table 2, the mean THI scores decreased from 53.3 at baseline to 25.8 post-treatment, reflecting an average reduction of 27.5 points (a 51.6% relative improvement; *p* < 0.001). Similarly, mean TFI scores improved from 54.0 to 27.1, corresponding to a 26.9 point reduction (49.8% relative improvement; *p* < 0.001). The absolute change is calculated as follows:

(5) $$\mathrm{Absolute}\;\mathrm{Change}\;(\Delta )=X_{\mathrm{baseline}}-X_{\mathrm{post}}$$

The relative change is as follows:

(6) $$\%\;\mathrm{Relative}\;\mathrm{Change}=\left(\frac{X_{baseline}-X_{post}}{X_{baseline}}\right)\times 100.$$

When analyzed by stimulus subtype, both groups demonstrated comparable effectiveness. The EAERR group exhibited a 51.4% improvement in THI and 50.7% improvement in TFI scores (all with *p* < 0.001). The EAEGT group showed a 51.8% THI improvement and 48.7% TFI improvement (all with *p* < 0.001).

Paired *t*-tests were applied to tinnitus-related distress reductions assessed with THI and TFI. The null hypothesis was that both stimuli provide equal mean reduction at the 5% significance level. No statistically significant differences in treatment response were observed for the group of all participants (*p* = 0.84). However, both scales provide statistically different mean improvement for the EAERR and EAEGT groups (*p* < 0.001).

**Figure 3:**
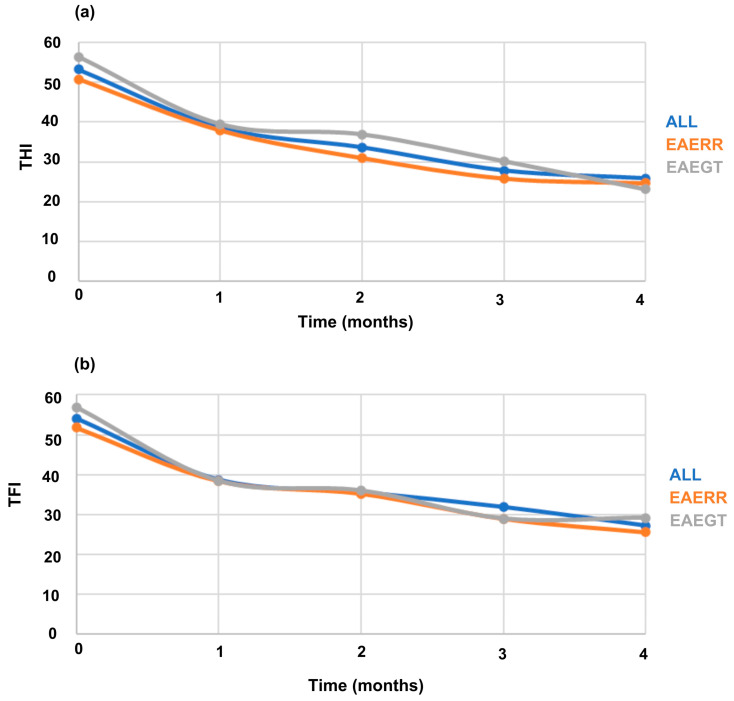
Monthly evolution of THI (**a**) and TFI (**b**) across the four-month EAE therapy in all participants, and in the EAERR and EAEGT groups.

Figure 3 shows the progressive reduction in THI and TFI scores over the four-month EAE program for all participants (blue), the EAERR group (orange), and the EAEGT group (gray).

### 3.2. Comparative Time Efficiency

To further assess treatment efficiency, absolute and relative improvements were normalized to the total listening time. All participants completed approximately 120 h of sound exposure across the four-month protocol. As shown in Table 3, the overall cohort achieved a 27.5-point reduction in THI and a 26.9-point reduction in TFI, corresponding to 51.6% and 49.8% relative improvement, respectively. When expressed as improvement per 10 h of therapy, this equated to 2.3 THI points and 2.2 TFI points, or 4.3% and 4.1% in relative percentages of change per 10 h.

Both stimuli provided comparable time-efficiency profiles. The EAERR subgroup showed a 26.1-point THI reduction (51.4% improvement) and 26.2-point TFI reduction (50.7% improvement), reflecting 2.2-point and 2.2-point improvement per 10 h, respectively. Likewise, the EAEGT subgroup achieved a 29.2-point THI reduction (51.8% improvement) and 27.7-point TFI reduction (48.7% improvement), corresponding to 2.4 THI points and 2.3 TFI points per 10 h. Both stimuli provide equivalent therapeutic benefits relative to treatment duration.

**Figure 4:**
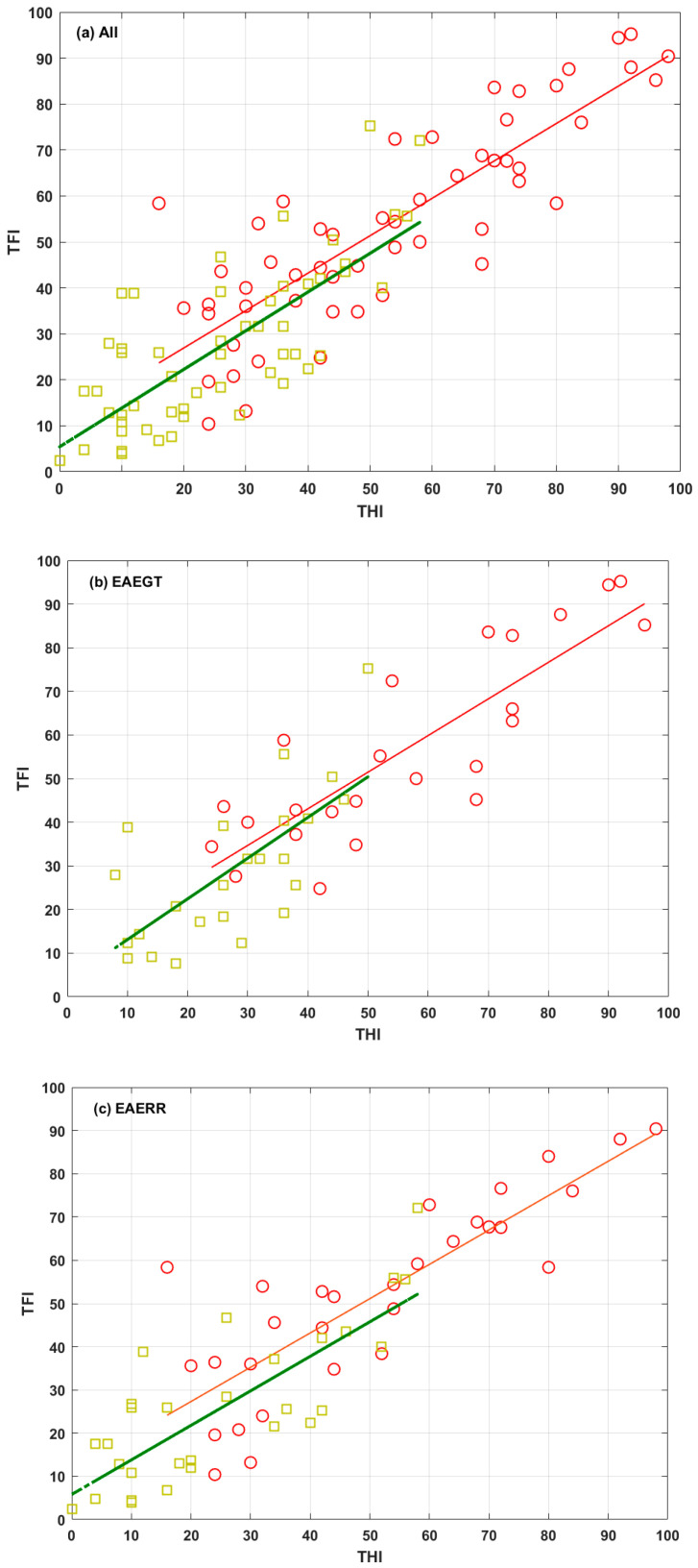
Scatter plots of TFI versus THI for the three groups: (**a**) All, (**b**) EAEGT, and (**c**) EAERR. Red circles correspond to (THI, TFI) values at baseline condition. Red lines are the corresponding linear fits. Green squares correspond to (THI, TFI) values after the EAE treatment. Green lines are the corresponding linear fits.

As reported previously [17], THI and TFI changes were closely aligned in all cases. To emphasize this close alignment, Figure 4 shows scatter plots of the scores of both questionnaires, pre (red) and post (green) EAE treatment, for the three groups (all, EAEGT, and EAERR). The corresponding linear fits are also shown.

### 3.3. Comparison with Standard TRT Outcomes from Literature

To contextualize the efficiency of the EAE protocol, a comparative analysis was conducted, including only tinnitus interventions combining counseling and sound therapy, aligned with the neurophysiological model. The THI was used as the primary outcome indicator given its extensive validation and widespread use in clinical tinnitus research.

Studies were eligible for inclusion when they reported baseline and post-treatment THI values, enabling calculation of absolute and relative improvement. Because treatment duration and daily sound-exposure requirements vary considerably across protocols, outcomes were standardized per 10 h of sound therapy exposure to provide a unified metric of treatment’s time efficiency.

The primary source of comparison studies was the review “The State of the Art of Sound Therapy for Subjective Tinnitus in Adults” [18]. Specifically, only those studies within this review that implemented TRT and reported complete THI data were extracted. Additional counseling-plus-sound therapy studies meeting these same criteria were also incorporated where appropriate. Table 4 summarizes all eligible studies, detailing baseline THI values, post-treatment scores, absolute and relative reductions, and normalized efficiency indices.

Across the literature, conventional TRT typically achieves reductions of approximately 20–25 THI points after >2000 h of sound exposure. When normalized per 10 h, these improvements yield modest efficiency values (all <0.25). In contrast, the EAE protocol demonstrated a mean THI reduction of ≈25 points (27.5 in this study) with only 120 h of sound exposure, corresponding to substantially greater improvement per unit of treatment time relative to TRT studies reported in the literature (2.3 in this study and >1.9 in the rest), being a significantly higher value per unit of time than conventional TRT.

Although methodological differences between studies preclude direct statistical comparison, the standardized time-efficiency framework applied here provides a clinically meaningful perspective for assessing treatment burden relative to therapeutic benefit. Within this context, the time efficiency of EAE appears to be substantially higher (on the order of approximately 20-fold) than the average reported for conventional TRT protocols. Nevertheless, it should be interpreted cautiously due to the heterogeneity of study designs and outcome timing.

## 4. Discussion

The present study evaluated both the clinical effectiveness and the time efficiency of EAE therapy for the management of chronic subjective tinnitus. In addition to assessing therapeutic outcomes, this work compared the efficiency of EAE relative to established tinnitus interventions grounded in the same neurophysiological framework, particularly TRT.

### 4.1. Clinical Outcomes

EAE produced clinically relevant and statistically significant reductions (*p* < 0.05) in tinnitus severity, with mean improvements of ~52% in THI and ~50% in TFI scores over four months.

Both stimuli demonstrated high responder rates and comparable efficacy. Notably, non-responders were minimal (8% in the EAERR group and 3% in the EAEGT group), highlighting strong treatment acceptability and response consistency.

Dropout rates differed between groups (EAEGT: 21%; EAERR: 2%). One possible explanation is that participants in the EAEGT group presented higher baseline tinnitus severity (THI: 62.5; TFI: 58) compared with those who dropped out from the EAERR group (THI: 24; TFI: 26), which may have influenced perceived need or motivation for treatment. Another hypothesis is related to stimulus characteristics: the sequential EAEGT stimulus has lower spectral density and provides less partial masking, which may have led some participants to perceive their tinnitus more prominently during listening and discontinue therapy.

On the other hand, the EAEGT group, which had slightly better baseline hearing thresholds, showed a larger absolute THI reduction (29.2 points vs. 26.1 in EAERR), but this difference was smaller in relative terms (51.8% vs. 51.4%). A similar trend was found for TFI, where EAEGT achieved a greater absolute reduction (27.7 vs. 26.2 points), while relative improvement favored EAERR (50.7% vs. 48.7%). No statistically significant differences emerged between groups, indicating that both continuous and sequential EAE stimuli yielded comparable and robust therapeutic outcomes.

### 4.2. Time-Efficiency and Adherence Advantages

A key advantage of the EAE protocol is its substantially reduced treatment burden compared with conventional TRT. While TRT typically requires 6–8 h of daily sound exposure for 12–24 months, EAE achieved comparable relative improvements with only one hour per day for four months (120 total hours vs. >2000 h. Normalized to 10 h of listening time, EAE achieved approximately 4.3% improvement per 10 h. Such an early, perceptible benefit likely supported the high adherence observed by offering a clinically meaningful change with a modest and manageable daily effort. The feasibility of using standard consumer headphones rather than specialized devices further supports scalability and clinical accessibility.

Mechanistically, EAE is grounded in the same neurophysiological principles as TRT, combining directive counseling with tailored sound enrichment to promote habituation. The individualized spectral shaping of the auditory stimulus aims to compensate for hearing deficits and rebalance auditory input, potentially accelerating adaptive plasticity and reducing tinnitus salience. This rationale aligns with contemporary frameworks emphasizing enriched stimulation to counteract maladaptive cortical reorganization. The favorable time-efficiency profile observed for EAE may also reflect the advantage of reduced sound-exposure demands, which could minimize auditory fatigue and limit potential excitotoxic effects associated with prolonged acoustic stimulation [29,30]. Nevertheless, the possibility that shorter protocols appear disproportionately efficient, compared to the differing follow-up durations among TRT studies, suggests that these efficiency metrics should be interpreted cautiously.

### 4.3. Comparison with Published TRT Literature

To contextualize the present findings, EAE outcomes of this cohort were compared with TRT studies identified in “The State of the Art of Sound Therapy for Subjective Tinnitus in Adults” [18] and additional trials meeting inclusion criteria (counseling + sound therapy; baseline and post-treatment THI available). Across these studies, TRT typically produces THI reductions of approximately 20–25 points over 12–24 months of treatment, requiring 6–8 h of daily sound exposure—representing more than 2000 cumulative therapy hours. In contrast, EAE achieved a greater mean THI reduction (27.5 points) with only 120 h of listening time. When normalized per 10 h of therapy, EAE demonstrated roughly a 20-fold higher time efficiency than the average reported for TRT protocols.

It is acknowledged that tinnitus improvement often occurs more rapidly during the initial treatment phase, after which progress stabilizes. This pattern may favor shorter protocols when calculating efficiency per hour, potentially introducing temporal bias. Nonetheless, given the magnitude of the efficiency differential observed (>20-fold), the findings suggest that structured, shorter-duration sound therapy may be sufficient to induce clinically meaningful habituation while enhancing adherence, reducing treatment burden, and reducing potential undesirable effects, such as auditory fatigue or excitotoxicity. In fact, no participant reported adverse effects, such as discomfort, listening fatigue, or ear fullness, likely due to the low playing intensity (always below their tinnitus sensation level) and the short exposition time (one hour). These results support further investigation into whether long-term, high-dose sound exposure is actually necessary for habituation-based tinnitus therapy.

Although CBT and mindfulness are relevant tinnitus treatments, they were not included in the present comparison because they do not incorporate sound therapy and therefore cannot be assessed using listening-time or time-efficiency metrics. Their effects (≈10–15-point THI reduction for CBT [6] and ≈11–13 points for mindfulness [31]) cannot be normalized per treatment hour. Since our objective was to compare EAE with interventions grounded in the neurophysiological model—that is, therapies combining counseling and sound stimulation—the analysis was appropriately restricted to TRT and related counseling-plus-sound protocols where treatment duration can be quantified.

### 4.4. Clinical Implications

EAE demonstrated tinnitus improvements comparable to those typically reported for TRT, but with a dramatically reduced therapeutic burden, requiring over 90% less total sound-exposure time. This shorter, structured format was associated with high adherence and strong clinical viability, positioning EAE as a more practical option for routine care. Because the intervention uses standard consumer headphones and no specialized devices, it is low-cost, accessible, and feasible for public healthcare systems, where long-duration TRT protocols are often impractical. This may contribute to supporting the inclusion of therapies based on the neurophysiological model in clinical guidelines for tinnitus management, something that is not currently the case in the vast majority of countries.

The efficacy observed across different tinnitus profiles (including bilateral and unilateral cases and varying degrees of hearing loss) supports broad clinical applicability. Moreover, the simple delivery format and patient-directed listening protocol make EAE particularly suitable for telemedicine and hybrid care models, expanding access without compromising therapeutic benefit. Together, these advantages suggest that EAE represents a scalable, patient-centered alternative for managing chronic tinnitus in modern clinical environments.

### 4.5. Limitations of the Study

This study presents several limitations that should be acknowledged. The sample size, although adequate for a feasibility analysis, was relatively small and may limit the generalizability of the findings. The absence of a control group or a TRT comparison arm prevents direct evaluation of EAE against established interventions. Adherence was monitored through self-reported data rather than device-logged measures, which may introduce reporting bias. Additionally, follow-up was limited to four months, preventing assessment of the long-term stability of therapeutic effects. These methodological constraints should be taken into account when interpreting the results.

## 5. Conclusions

EAE produced clinically meaningful and statistically significant reductions in tinnitus severity, achieving approximately 50% improvement in THI and TFI scores following a protocol of one hour per day for four months. Both continuous and sequential EAE stimuli yielded similar therapeutic outcomes, with very low rates of non-responders and high adherence, supporting the reliability and consistency of the intervention across different patient profiles.

A key advantage of EAE is its markedly reduced therapeutic burden. Despite requiring over 90% less sound-exposure time than traditional TRT, EAE achieved comparable or greater improvements. When normalized relative to treatment time, EAE demonstrated substantially superior time efficiency, suggesting that shorter, structured auditory stimulation may be sufficient to promote habituation while enhancing adherence and minimizing treatment fatigue. The protocol’s simplicity, use of standard consumer devices, and compatibility with remote delivery support its clinical scalability and make it particularly suitable for public healthcare systems and telemedicine frameworks.

Although the absence of a control group and long-term follow-up limits definitive comparison with other treatments, the findings indicate that EAE is a practical, accessible, and time-efficient alternative to conventional TRT. Future research should include multicenter, long-term randomized trials to confirm these results, assess sustained benefit, and evaluate implementation across different clinical settings. In parallel, advances such as AI-driven sound personalization, automated adherence monitoring, and adaptive real-time stimulus adjustment offer promising opportunities to further optimize EAE and facilitate its integration into digital health and tele-audiology ecosystems. These developments may enhance large-scale feasibility and support broader incorporation of neurophysiologically based sound therapies into evidence-based tinnitus management.

## Figures and Tables

**Table 1:** Number of responding subjects in each sound therapy group, along with the gender, age, tinnitus laterality, duration, frequency, and initial THI and TFI score. M: mean; SD: standard deviation.

	All (*n* = 54)	EAERR (*n* = 30)	EAEGT (*n* = 24)
Gender	Male: 33 (61%)	Male: 20 (67%)	Male: 13 (54%)
	Fem: 21 (39%)	Fem: 10 (33%)	Fem: 11 (46%)
Age (years)	M: 52.2	M: 53.3	M: 50.9
	SD: 11.7	SD: 12.4	SD: 10.8
Tinnitus laterality (Bilateral = B; Right ear = R; Left ear = L)	B: 26 R: 13 L: 15	B: 15 R: 5 L: 10	B: 11 R: 8 L: 5
Tinnitus duration (years)	M: 7.7	M: 8.2	M: 7.1
	SD: 10.4	SD: 11.5	SD: 9.1
Tinnitus Pitch (Hz)	M: 4385 SD: 2699	M: 5242 SD: 2888	M: 3313 SD: 2031
THI_baseline_	M: 53.3 SD: 22.6	M: 50.8 SD: 23.2	M: 56.4 SD: 22.0
TFI_baseline_	M: 54.0 SD: 21.8	M: 51.8 SD: 21.9	M: 56.9 SD: 21.7

**Table 2:** Baseline and post-treatment THI and TFI scores mean change, relative improvement, and *p*-values for the overall sample and by stimulus type.

Measure	Group	Baseline (Mean ± SD)	Post (Mean ± SD)	Δ (Mean ± SD)	Relative % Improvement	*p*-Value
	All	53.3 ± 22.6	25.8 ± 15.5	27.5 ± 16.2	51.6%	*p* < 0.001
THI	EAERR	50.8 ± 23.2	24.7 ± 17.5	26.1 ± 16.3	51.4%	*p* < 0.001
	EAEGT	56.4 ± 22.0	27.2 ± 12.6	29.2 ± 16.2	51.8%	*p* < 0.001
	All	54.0 ± 21.8	27.1 ± 17.2	26.9 ± 15.7	49.8%	*p* < 0.001
TFI	EAERR	51.8 ± 21.9	25.5 ± 17.6	26.2 ± 13.9	50.7%	*p* < 0.001
	EAEGT	56.9 ± 21.7	29.2 ± 16.8	27.7 ± 17.9	48.7%	*p* < 0.001

**Table 3:** Absolute and relative improvement in THI and TFI scores and time-efficiency ratios after 4-month EAE therapy for the overall sample and by stimulus type.

	THI					TFI			
	Listening Time (h)	Δ	Δ/10 h	% Δ	% Δ/10 h	Δ	Δ/10 h	% Δ	% Δ/10 h
All	120 h	27.5	2.3	51.6%	4.3%	26.9	2.2	49.8%	4.1%
EAERR	120 h	26.1	2.2	51.4%	4.3%	26.2	2.2	50.7%	4.2%
EAEGT	120 h	29.2	2.4	51.8%	4.3%	27.7	2.3	48.7%	4.1%

**Table 4:** Sample size (*n*), treatment duration, total listening hours, baseline THI, relative THI improvement, improvement per 10 h, absolute THI change (ΔTHI), and ΔTHI per 10 h. Only studies using TRT or counseling-plus-sound therapy (EAE and Masking) with baseline and post-treatment THI data were included.

Therapy	*n*	Duration (Months)	Total Listening (Hours)	THI_baseline_	Relative Improvement (%)	% Relative/ 10 h	ΔTHI	ΔTHI/ 10 h	Reference
EAE (this study)	54	4	120 h (1 h/d)	53.3	51.6%	4.3%	−27.5	2.3	
EAE	123	4	120 h (1 h/d)	53.4	45.5%	3.8%	−24.3	2.0	[9]
EAE	80	4	120 h (1 h/d)	49	46.9%	3.9%	−23	1.9	[19]
Tinnitus Masking	46	18	3240 h–4320 h (6–8 h/d) *	53.7 **	21.6%	0.07%	−11.6	0.04	[20]
Smart TRT	42	3	540 h (6 h/d)	46.9	49.7%	0.92%	−23.3	0.43	[21]
TRT	42	3	540 h (6 h/d)	50.5	25.5%	0.47%	−12.9	0.24	[21]
TRT	34	18	4320 h (8 h/d)	37.8	16.1%	0.03%	−6.1	0.07	[22]
Multicolor TRT	20	6	1080 (6 h/d)	44.7	44.0%	0.40%	−19.65	0.18	[23]
White Noise TRT	20	6	1080 (6 h/d)	48.5	40.5%	0.18%	−19.65	0.18	[23]
TRT	19	18	5637 h (10.4 h/d)	46.7	63.0%	0.11%	−29.4	0.05	[24]
TRT	14	18	4320 h (8 h/d)	47	10.9%	0.03%	−5.14	0.01	[25]
TRT	33	12	2880 h (8 h/d)	≈62	66.1%	0.22%	−≈41	0.14	[26]
TRT	45	18	2880 h (8 h/d)	57.8	48.4%	0.17%	−28	0.10	[27]
TRT	48	18	3240 h–4320 h (6–8 h/d) *	51.4 **	58.6%	0.18%	−30.2	0.09	[20]
TRT	68	12	2880 h (8 h/d)	56.2	26.7%	0.09%	−15	0.05	[28]

* Not specifically stated in the article. We assume the listening hours follow the standard TRT protocol. ** Present the data stratified by subgroups. A weighted mean was calculated based on the sample.

## Data Availability

The data are not publicly available due to the confidentiality clause of the informed consent form.
